# Editorial: Carotid body: a new target for rescuing neural control of cardiorespiratory balance in disease

**DOI:** 10.3389/fphys.2015.00181

**Published:** 2015-06-29

**Authors:** Rodrigo Del Rio, Rodrigo Iturriaga, Harold D. Schultz

**Affiliations:** ^1^Laboratory of Cardiorespiratory Control, Center for Biomedical Research, Universidad Autónoma de ChileSantiago, Chile; ^2^Laboratory of Neurobiology, Facultad de Ciencias Biológicas, Pontificia Universidad Católica de ChileSantiago, Chile; ^3^Department of Cellular and Integrative Physiology, University of Nebraska Medical CenterOmaha, NE, USA

**Keywords:** carotid body, heart failure, sleep apnea, hypertension, autonomic function, sympathetic nervous system, insulin resistance

The carotid body (CB) is considered the main arterial chemoreceptor. Interestingly, the CB respond to a large repertoire of stimuli that can elicit a reflex cardiorespiratory response. For this reason, the pathophysiological role played by alterations in CB function during disease conditions is under intense research.

Since its discovery, the CB has been related almost exclusively to its pivotal role in adjusting ventilatory and cardiovascular function during acute or chronic alterations on blood gases (i.e., hypoxia and hypercapnia, sustained chronic hypoxia). However, recent advances has contribute to deciphere the role played by the CB in the progression of highly prevalent diseases such as hypertension, heart failure and insulin resistance. Indeed, elegant studies showing the effect of CB neurotomy in pathophysiology have unveiled a key role of these arterial chemoreceptors in the development of autonomic imbalance, respiratory disturbances, systemic inflammation, and impairment of glucose metabolism (Fitzgerald, 2014). Therefore, targeting the CB rises as a novel therapeutic strategy to improve disease outcome. However, the mechanisms involved in the alterations of CB function in pathophysiology are not completely understood. Indeed, classical pharmacotherapy intended to normalize CB function may be hard to establish since several cellular pathways are involved in the CB dysfunction. Augmented levels of CB neuromodulators such as angiotensin II, endothelin-1, pro-inflammatory cytokines, cyclic nucleotides, hydrogen sulfide, carbon monoxide and free radicals had all been related to CB chemosensory facilitation (Iturriaga et al., 2014; Prabhakhar and Joyner, 2015). Indeed, it has been proposed that the enhanced CB chemoreflex drive induces central nervous system (CNS) plasticity.

In experimental chronic heart failure (CHF), the CB chemosensory activity is tonically elevated leading to sympatho-excitation and destabilization of breathing. Marcus et al. (2014) reviewed the contribution of the CB on the respiratory-sympathetic coupling in CHF and its role in the development of oscillatory breathing patterns and enhanced renal sympathetic nerve activity. The elimination of the CB afferents totally normalized breathing patterns and reduced sympathetic outflow suggesting that the CB plays a pivotal role in the progression of cardio-respiratory dysfunction during CHF.

It has been shown that exposure to chronic intermittent hypoxia (CIH), one of the main features of obstructive sleep apnea syndrome (OSA), induces a potentiation of CB chemosensory activity (Iturriaga et al., 2014). It has been proposed that episodic hypoxia during CIH induces CB sensory plasticity increasing neural afferent discharges to brainstem areas related to cardiorespiratory control (Fung, 2014; Iturriaga et al., 2014; Prabhakhar and Joyner, 2015). The hyperactivation of the CB-mediated chemoreflex following CIH leads to hypertension. The cellular mechanisms underlying the CB sensitization during CIH are not completely known; however, it is well accepted that endothelin, angiotensin peptides, pro-inflammatory cytokines and oxidative stress are all involved in the sensory potentiation of the CB (Iturriaga et al., 2014). Fung (2014) reviewed the concept of a local renin-angiotensin system (RAS) within the CB, which could be promoting functional adjustments of the CB function during CIH. Interestingly, the RAS in the CB displayed a marked upregulation following CIH suggesting a plausible role during CB sensory plasticity. Therefore, specific blockers of the RAS should be of benefit in the control of CB-mediated hypertension during CIH. Accordingly, Diogo and Monteiro (2014) provide a comprehensive review of the different treatments and strategies to reduced blood pressure in both animal models and in humans with OSA. Intriguingly, there is a still lack of a unique treatment to control OSA-related hypertension. Therefore, future studies are needed.

Maintenance of glucose homeostasis is a key process for the proper metabolic functions of several tissues across the body. Accordingly, alterations on glucose sensing could severely impact the development of pathological conditions, especially those linked to sympatho-excitation (Conde et al., 2014; Gao et al., 2014). Nevertheless, the idea that CB chemoreceptor cells respond to changes in arterial glucose levels is still a matter of debate. However, Gao et al. (2014) provide compelling evidence showing that low glucose induced increases in intracellular Ca^2+^ levels and catecholamine release in isolated CB chemoreceptor cells. Furthermore, they described that glucose-sensing pathways in the CB are not shared by the oxygen sensing mechanisms by which the CB respond to low arterial oxygen levels (Gao et al., 2014). In addition, Conde et al. (2014) described the pivotal role of the CB in the development of insulin resistance and the consequent progression into type 2 diabetes. Furthermore, they proposed targeting the CB as a potential therapeutic strategy to improved glucose metabolism. In addition, Conde et al. (2014) proposed chronic caffeine intake as a plausible strategy intended to normalize CB function in insulin resistance and type 2 diabetes due to the well documented effects of caffeine on adenosine receptors within CB chemoreceptor cells. Unfortunately, there are no human studies showing a beneficial effect of caffeine intake during the transition from insulin resistance to diabetes. Future studies should be focused on the therapeutic effect of caffeine on the CB-mediated glucose sensing impairment during diabetes.

The immune system is the barrier of defense during pathogen infection. Recently, neuro-immunomodulation of the inflammatory process has been point out as a central step in the immune response. In this context, activation of peripheral sensory afferents by pro-inflammatory cytokines is of interest to understand the immunosensory modulation (Fernandez et al., 2014). Despite the evidence showing cytokine receptor expression on vagal paraganglia, the CB rises as a plausible sensor of inflammatory status since the CB displays cytokine receptors in chemoreceptor cells and that upon activation, elicit a reflex response intended to regulate the inflammatory response. Fernandez et al. (2014) reviewed the contribution of the CB on sepsis progression and the role of the CB on exacerbating the immune defense. Contrary to the above mentioned pathologies (heart failure, OSA, diabetes), during sepsis, activation of the CB appears to be helpful in the inflammatory process since it will induce corticoid release by the adrenal gland in a mechanism linked to an increase in sympathetic outflow. Thus, stimulation of the CB may be a suitable tool to improve the outcome during sepsis and infectious diseases.

Understanding the CB chemosensory process, regardless of it's involvement in pathophysiological events, is extremely relevant to improve knowledge in the field. The mechanisms that govern the CB chemoreceptor response to hypoxia are not fully understood. Nunes et al. (2014) reviewed the contribution of cyclic adenosine monophosphate (cAMP) to the CB chemosensory transduction process. Despite the fact that cAMP is considered mainly a metabolic product, its ability to modulate G-coupled receptors and change intracellular Ca^2+^ levels in CB chemoreceptor cells (or petrosal afferents) make this nucleotide an interesting candidate to be considered as a CB chemosensory modulator. In the same context of finding new pathways involved in the CB function, Mazzatenta et al. (2014) showed novel data regarding the role of galanin, a 30-aminoacid neuropeptide, in CB neurogenesis. Interestingly, the expression of this peptide in the CB seems to correlate with age. They showed that “old CBs” displayed less galanin compared to “young CBs.” Therefore, it is plausible that galanin contributes to the loss of CB sensitivity during aging.

Also important in the CB-mediated chemoreflex is the mechanism by which the afferent information travels to the CNS. Retamal et al. (2014) reviewed the importance of the petrosal ganglion neurons in the CB chemotransduction process. Historically, this group of neurons was normally described as part of the “wiring” connection between the CB and the CNS. However, evidence from other sensory ganglia suggests that neuronal-to-glial cell communication can modulate sensory information. Thus, glial cells may modulate the excitatory or inhibitory status of petrosal ganglion neurons. The outcome would be an increase or decrease of the CB afferent input to the CNS. This exciting hypothesis deserves future investigations.

The CB undergoes structural and functional changes during development and in response to chronic sustained hypoxia. Taking into account the small size of the CB (≈1 mm^3^ in humans), novel techniques are always required to improve experimental approaches. Guidolin et al. (2014) provide an interesting new method to study structural changes in the CB. Using fractal analysis they study the extracellular matrix composition in fixed and stained sections from the CB. They found that fractal analysis of CB sections is a suitable tool to obtained quantitative data of the extracellular components present in the CB. This technique could be useful in the study of CB structural remodeling in disease conditions.

In summary, this collection of works provides a useful and timely update in the field of CB chemoreception and its contribution on the development and progression of several pathologies. Improving the knowledge on the role of the CB in health and disease will promote new avenues in the understanding of the CB-mediated chemoreflex.

## Conflict of interest statement

The authors declare that the research was conducted in the absence of any commercial or financial relationships that could be construed as a potential conflict of interest.
